# Role of Oral Bacteria in the Development of Oral Squamous Cell Carcinoma

**DOI:** 10.3390/cancers12102797

**Published:** 2020-09-29

**Authors:** Qinyang Li, Yao Hu, Xuedong Zhou, Shiyu Liu, Qi Han, Lei Cheng

**Affiliations:** State Key Laboratory of Oral Diseases & West China School of Stomatology & National Clinical Research Center for Oral Diseases, Sichuan University, Chengdu 610041, China; 2016151642104@stu.scu.edu.cn (Q.L.); huyao93@stu.scu.edu.cn (Y.H.); zhouxd@scu.edu.cn (X.Z.); liusy9307@163.com (S.L.)

**Keywords:** Oral squamous cell carcinoma, oral bacteria, inflammation

## Abstract

**Simple Summary:**

Oral squamous cell cancer (OSCC) is still one of the major malignant tumors of the head and neck region with dissatisfactory survival rate. Recently, based on the high-throughput sequencing technology, OSCC has been verified a close relationship with oral bacteria. Our review aims to summarize these findings and raise our perspectives. We conclude that different oral bacteria show distinct alterations in the abundance and a certain combination of various bacteria might possibly be markers for OSCC diagnosis. Besides, oral bacteria such as *Porphyromonas gingivalis* and *Fusobacterium nucleatum* can participate in most cancer-promoting pathways to assist OSCC development. Therefore, oral bacteria may be a target to provide potential methods for early diagnosis and more effective treatments.

**Abstract:**

Oral squamous cell carcinoma (OSCC) is an invasive epithelial neoplasm that is influenced by various risk factors, with a low survival rate and an increasing death rate. In the past few years, with the verification of the close relationship between different types of cancers and the microbiome, research has focused on the compositional changes of oral bacteria and their role in OSCC. Generally, oral bacteria can participate in OSCC development by promoting cell proliferation and angiogenesis, influencing normal apoptosis, facilitating invasion and metastasis, and assisting cancer stem cells. The study findings on the association between oral bacteria and OSCC may provide new insight into methods for early diagnosis and treatment development.

## 1. Introduction

Oral cancer is one of the major malignant tumors of the head and neck region, causing great mortality and morbidity [1,2]. According to the World Health Organization (WHO), there are around 657,000 new cases of oral cavity and pharyngeal cancers each year, with more than 330,000 deaths. Oral squamous cell carcinoma (OSCC), an invasive epithelial neoplasm with different degrees of differentiation, accounts for about 90% of oral cancer. It starts with the accumulation of genetic mutations and specific genetic variations in oncogenes and suppressor genes [3]. The high-risk areas are the floor of the mouth and the ventrolateral tongue, while the low-risk regions lie in the palatal mucosa and the tongue dorsum [4].

The key to OSCC management is early diagnosis and treatment. Targeting pre-malignant oral diseases has been regarded as a possible strategy for the early diagnosis of at-risk and high-risk patients, but it remains difficult to diagnose clinically [5,6]. The most common treatment for OSCC is surgical resection, but radiotherapy and chemotherapy are used preoperatively and postoperatively to reduce difficulties in surgical removal or eliminate remaining cancer cells [7,8]. An epidermal growth factor receptor (EGFR) antibody has been developed, aimed at decreasing the over-expression of EGFR, which has been associated with the poor prognosis of OSCC patients [9]. However, despite more diverse and advanced treatment options, the five-year survival rate remains below 50% [10,11,12].

OSCC is influenced by various factors, including tobacco smoking, alcohol abuse, HPV (human papillomavirus) infection, male gender, economic status, dietary habits, and oral hygiene [13,14,15,16]. Lately, studies have verified a close link between OSCC and oral bacteria, which may provide a fresh view and new potential targets for OSCC diagnosis and treatment [17,18,19].

The oral bacteria are the second-largest human-associated microbial community, only exceeded by bacteria in the gut, and constitute a unique micro-ecology in the oral cavity. More than 700 bacterial species survive in it, along with archaea, fungi, and viruses. In the normal oral cavity, bacteria react with each other but maintain a good balance so that oral health can be achieved. However, when something breaks the balance, dysbiosis occurs and the healthy, balanced oral ecosystem is destroyed. Oral pathogens take advantage of the imbalance, leading to diseases such as caries and periodontal disease [20,21]. Lately, many studies have demonstrated that oral bacteria could also be important during the process of OSCC by exploring changes in the abundance of oral bacteria and mechanisms that might participate in the development of cancer [22,23]. Bacterial compositional differences have been demonstrated among cancer patients, normal individuals, and pre-cancer patients [22,23]. Other studies have verified the potential mechanisms by which oral bacteria affect the development of OSCC, including speeding up cell proliferation, inhibiting apoptosis, and improving tumor invasion and metastasis [24].

This review summarizes the compositional changes in bacteria in OSCC patients and the possible mechanisms associated with oral cancer development, hoping to reveal the hidden link between certain oral bacteria and OSCC and provide new sight into the prevention, prediction, and treatment of oral cancer.

## 2. Compositional Variations in Oral Bacteria in OSCC

Over the past 10 years, several studies have taken fresh perspectives on the variations in oral bacteria associated with OSCC and their analyses showed similarities and differences. Using 16S rRNA amplicon sequencing to compare oral bacterial DNA isolated from cancer patients and normal subjects, the findings revealed comprehensive relationships between OSCC and oral bacteria [25].

Pathogenic periodontal bacteria are a group of bacteria associated with periodontal diseases and contribute to an inflammatory state, which may induce DNA damage in epithelial cells in cancer progression [22]. Periodontal pathogenic bacteria have been associated with a higher risk for OSCC and *Fusobacterium*, *Peptostreptococcus*, *Filifactor*, *Parvimonas*, *Pseudomonas*, *Campylobacter*, and *Capnocytophaga* were reported in significantly high abundance in OSCC patients. Moreover, proinflammatory substances secreted by periodontal pathogenic bacteria such as lipopolysaccharide (LPS) were enriched in cancer samples [26]. Among the bacteria, *Fusobacterium* showed the most verified alterations in cancerous lesions [23,27,28]. In a group of cancer patients, *Fusobacterium* showed a significant increase in abundance, especially in stage 4 patients [23,27,28]. Moreover, some research suggested that *Fusobacterium* might be an essential part of the bacterial impact on OSCC since it had the ability to co-adhere with other species and form an association network centered around itself in patients of the cancer group [23,29]. *Peptostreptococcus*, *Parvimonas,* and *Campylobacter* are anaerobic bacteria reported to participate in the colorectal cancer process [30,31]. Two other genera, *Filifactor* and *Capnocytophaga*, have been closely linked to lung cancer [32,33]. Nevertheless, how they are involved in OSCC is still unclear, thus, further studies are required. *Pseudomonas* has not been linked to cancer yet, but it possesses virulence factors such as LPS and flagella, which play roles in carcinogenesis by counteracting host defenses and causing direct damage to host tissues [34]. In addition, *P. aeruginosa* could injure epithelial cells by triggering DNA strand breaks [35]. Therefore, we believe that the ability of periodontal pathogens, particularly *Fusobacterium nucleatum*, to aggregate and induce inflammation, which may cause DNA damage to epithelial cells leading to cancer progression, might account for the compositional change in bacteria in tumor sites.

Periodontal pathogenic bacteria appeared to be positively associated with OSCC, however, other bacteria have shown different changes. *Firmicutes* (especially *Streptococcus*) and *Actinobacteria* (especially *Rothia*) comprised a smaller proportion of the bacteria in OSCC tissues and were enriched in the healthy group [23,29]. *Streptococcus* was linked to *F. nucleatum* in some aspects [36,37]. On one hand, *Streptococcus* is an early colonizer but *F. nucleatum* is a transitional bacteria between early and late colonizers in the OSCC process, with an ability to co-aggregate [36]. On the other hand, *Streptococcus* could attenuate the proinflammatory responses of oral epithelial cells induced by *F. nucleatum* [37]. Moreover, *Firmicutes* and *Actinobacteria* both were confirmed to have a negative correlation with oral pre-cancer, suggesting that *Firmicutes* and *Actinobacteria* may be altered early in cancer development [18,29,38]. Thus, we believe that *Firmicutes* and *Actinobacteria* are sensitive to cancer-related circumstances, and a remarkably decreased proportion of these bacteria along with an increased proportion of *F. nucleatum* may indicate a pre-cancerous or cancerous state.

Besides *Firmicutes* and *Actinobacteria*, some research identified alterations in other oral bacteria in oral pre-cancerous conditions and differences between normal, pre-cancerous, and cancerous lesions. *Megasphaera micronuciformis*, *Prevotella melaninogenica*, and *Prevotella veroralis* were found more abundant in oral pre-cancer. *M. micronuciformis* seemed to be the best candidate for a specific biomarker as it was detected only in the pre-cancer group [39]. In addition, five genera, including *Bacillus*, *Enterococcus*, *Parvimonas*, *Peptostreptococcus,* and *Slackia*, displayed distinct differences in abundance between patients with epithelial precursor lesions and cancer patients [22]. It takes a long time to change from a pre-cancerous state to cancer. Therefore, bacterial changes at this stage may be a potential target for primary prevention. Furthermore, specific microbial combinations have been investigated as potential markers for OSCC diagnosis and demonstrated considerable accuracy [22,28]. In 2017, Zhao et al. claimed that a highly connected bacterial cluster of *Fusobacterium* comprising seven operational taxonomic units (OTUs) showed considerable predictive power in OSCC since the area under the receiver operating characteristic curve(AUC) reached 0.866 [23]. Another study conducted in 2018 by Yang et al. reported that a bacterial combination consisting of *Fusobacterium periodonticum*, *Streptococcus mitis*, and *Porphyromonas pasteri* had an AUC of 0.956 (95% CI: 0.925–0.986) in discriminating OSCC stage 4 from healthy controls [36]. Moreover, in 2017, Lee et al. concluded that *Bacillus*, *Enterococcus*, *Parvimonas*, *Peptostreptococcus*, and *Slackia* in saliva might be a group of potential biomarkers for OSCC diagnosis [22]. However, certain components of this marker are not officially accepted for diagnosis and prognosis despite its accuracy. Further studies are still needed to explore how oral bacteria act upon tumors and the converse situation.

The most recent 10-year research findings have reported changes in oral bacteria in OSCC (Appendix A Table A1). However, a complete consensus has not been reached. Different specimen types, controls, and methods might have contributed to the lack of consensus. Apart from these factors, disparate stages studied in OSCC may also be contributors to the inconsistent findings. In the progression from normal epithelial tissue to pre-malignant and subsequently cancerous lesions, the proportion of oral bacteria synchronously changes and the bacteria that adapt to or facilitate the tumor microenvironment at different phases become dominant. Here, we choose to introduce the so-called “drive-passengers” model to explain this phenomenon [40,41]. The “drivers” are defined as oral bacteria with pro-carcinogenic features such as the production of DNA-damaging compounds that might initiate oral cancer. For this reason, drivers emerge at early cancer stages. *Porphyromonas gingivalis* may be a driver since it can influence pathways related to DNA damage and directly damage DNA with its LPS, a potent inflammatory molecule with cancer-promoting properties [42,43]. Furthermore, in studies using high-throughput sequencing technology, a remarkable change in the abundance of *P. gingivalis* in OSCC has been reported and it seems to occur most in the very early stage of OSCC [28]. The passengers are said to be inhibited in a healthy oral state, but when dysbiosis occurs, such as in a cancer-related state, the passengers will have a competitive advantage in the tumor microenvironment. This kind of bacteria participates in the progression and promotion of tumors. From our point of view, *F. nucleatum* may be a vital passenger. The basis for this hypothesis is that *F. nucleatum* remains in low proportion in oral healthy cavities, but it increases significantly and seems to be the most prevalent in OSCC patients [44]. Moreover, it has been mentioned in regard to obvious compositional changes in oral cancer in most articles and reported to participate in the progression of OSCC by accelerating the proliferation of cancer cells and helping cancer cells to invade and metastasize [23,27,28,44,45,46,47,48]. Therefore, *F. nucleatum* could represent a signal for the malignant transformation of oral epithelial cells.

Moreover, compared with the inconsistent compositional alterations reported, functional changes in oral bacteria in OSCC may be more reliable. Several findings in the last two years demonstrated that the bacteria found within OSCC tissue were functionally proinflammatory [18,27,49], and LPS as well as peptidases, two proinflammatory substances from bacteria, were enriched in OSCC samples [26]. Meanwhile, a study that focused on the functional prediction of oral bacterial communities also revealed a potent enrichment in the genes involved in bacterial chemotaxis and flagellar assembly in cancer-related inflammation [22,29].

## 3. Mechanism of the Effect of Oral Bacteria on OSCC

OSCC is a malignant tumor originating from oral epithelial dysplasia. Furthermore, like other malignant tumors, it develops through the successive progression of various features and mechanisms such as the activation of oncogenes, the inhibition of cancer cell apoptosis, the promotion of invasiveness and metastasis, and changes in the tumor microenvironment [50,51,52]. The tumor microenvironment consists of the extracellular matrix, soluble molecules, and tumor stromal cells, which interact with a community of heterotypic cells [53]. Proinflammatory factors in the tumor microenvironment are part of the final outcome of a dynamic process that includes the chemotaxis of immune cells and the appearance of cytokines and chemokines in the tumor microenvironment, which are always over-expressed [54,55]. They cause a series of reactions that help tumor cells develop, and with the progression of the tumors, proinflammatory factors increase as well. Moreover, similar to the progression of other tumors, tumorigenesis, and tumor development is an on-going process, which means that cancer cells grow faster and show direct metastasis over wide ranges [19].

Recently, based on compositional alterations in oral bacteria during OSCC progression, numerous experiments have explored the probable mechanisms explaining how oral bacteria affect oral cancer. From a retrospective view of previous studies, although the bridges linked to cancer are diverse, the focus seems to be on the chronic inflammation involved in tumorigenesis and tumor progression [56,57]. Below are different mechanisms involved in the relationship between oral bacteria and OSCC, and among them, some mechanisms are associated with inflammation.

### 3.1. Cell Proliferation

**Figure 1:**
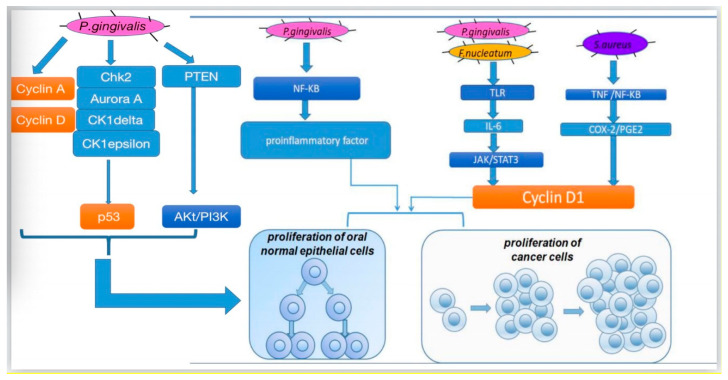
Mechanisms for how oral bacteria promote cell proliferation.

There are several pathways for different oral bacteria to promote cell proliferation (Figure 1). In vitro, oral bacteria such as *P. gingivalis* can lead to secondary impacts on the proliferation rate by modifying the expression levels of oncogenic-relevant α-defensin genes. Hoppe et al. incubated oral tumor cells with *P. gingivalis* and human α-defensins and observed a noticeable increase in tumor cell proliferation [58]. Similarly, another study conducted in the human squamous cell carcinoma cell line SCC-25 and primary human gingival keratinocytes revealed that, in both cells, *P. gingivalis* and its membrane fraction regulated some genes involved in the downstream signaling pathway of the proinflammatory active transcription factor NF-κB and some members of the MAPK family such as MAPK14 (p38), MAPK8 (JNK1), and NFKB1(p50), which participated in cancer proliferation and control [24]. Moreover, Kuboniwa et al. co-cultured 50% confluent human oral epithelial cell culture cells with *P. gingivalis* strains at 37 °C and 5% CO_2_ and demonstrated that *P. gingivalis* infection affected pathways related to cyclins, p53, and PI3K that exerted control over the cell cycle [43]. By regulating cyclin A and cyclin D, two nuclear proteins [59], *P. gingivalis* assisted gingival epithelial cells to progress through the G1 phase at a faster pace. By downregulating kinases such as Chk2, aurora A, CK1delta, and CK1epsilon to make p53, a suppressor that could arrest the cell cycle unstable and inactivate, *P. gingivalis* accelerated cell proliferation [60,61]. By downregulating PTEN, a lipid phosphatase that prevents the activation of Akt [62], *P. gingivalis* improved the levels of PI3K and phosphoinositide-dependent protein-serine kinase 1 (PDK1) activated by Akt, which promoted cell proliferation [63]. In addition, a study our team participated in found that when human oral keratinocyte cells were infected with inactivated *Staphylococcus aureus* in high-glucose Dulbecco’s modified Eagle’s medium, certain *S. aureus* genes upregulated COX-2 transcription, increased PGE2 production, and then induced higher expression of oral cancer-associated genes cyclin D1, which is associated with cell proliferation and growth regulation [64,65]. Combined with earlier studies on the relationship between OSCC and COX-2/PGE2, *S. aureus* is another likely vital bacterial candidate involved in cancer [66]. Other research in vivo mouse model and in vitro SCC-25 and CAL 27 human tongue SCC cell lines focusing on *P. gingivalis* and *F. nucleatum* observed the same results that by triggering Toll-like receptor (TLR) signaling, IL-6 production increased, and then activated STAT3, which induced important effectors such as cyclinD1, driving cancer cells to grow [47].

### 3.2. Cell Apoptosis

**Figure 2:**
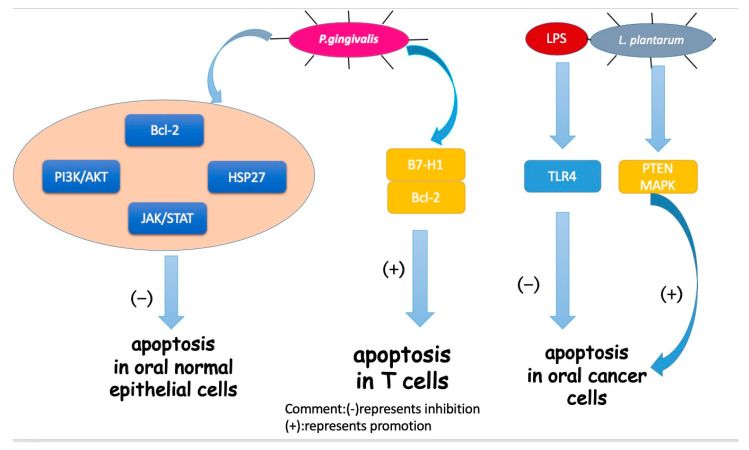
Mechanisms for how oral bacteria influence cell apoptosis.

Apoptosis is a certain programmed cell death process that is a vital part of the immune system [67]. It serves as a tumor suppressor in various cancer phases [68]. Based on recent studies, apoptosis in OSCC was associated with oral bacteria and different bacteria showed distinct impacts on apoptosis (Figure 2).

The representative oral pathogen, *P. gingivalis*, has been mostly studied. Not only can *P. gingivalis* inhibit the apoptosis of oral epithelial cells, but the bacteria can also induce the apoptosis of immune cells to help protect cancer cells from an immune attack. In oral epithelial cells, by increasing phosphatidylinositol 3-kinase/Akt signaling [69,70] and modulating Bcl-2 family proteins, *P. gingivalis* infection could improve the survival and proliferation of gingival epithelial cells (GECs), indirectly inhibiting intrinsic apoptosis. Moreover, a homolog of a conserved nucleoside-diphosphate-kinase (Ndk) family of multifunctional enzymes and secreted molecule of *P. gingivalis* inhibited apoptosis in GECs via phosphorylating HSP27 [71], a kind of heat shock protein that inhibits apoptosis [72]. Another pathway for *P. gingivalis* to inhibit apoptosis in GECs was through the manipulation of the JAK/STAT pathway, which controls the intrinsic mitochondrial cell death pathways [73]. In immune cells, Groeger et al. analyzed squamous carcinoma SCC-25 cells after infection with two virulent *P. gingivalis* strains (*P. gingivalis* strains W83 and ATCC 33277) and a commensal bacterium (*Streptococcus salivarius K12*). The results showed that only *P. gingivalis* could activate (B7-H1) receptors, which led to anergy and the apoptosis of activated T cells and helped cancer cells evade immune attack [74].

In the last few years, besides the mechanism mentioned above, triggered TLR signaling has been observed in oral tumorigenesis [75,76,77,78]. Although TLRs work mainly in immunity, some studies have reported their role in inhibiting apoptosis in cancer cells [79,80]. Lately, TLR2 and TLR4 were studied for their capacity to recognize different pathogens from bacteria. Recently, a group conducted a research based on the hypothesis that bacterial pathogens such as *P. gingivalis* and *F. nucleatum* may induce resistance to apoptosis by activating TLR2 [81]. They analyzed the expression of TLR-2 in clinical OSCC specimens and human OSCC-derived cell lines (HSC). They demonstrated that TLR2 was highly expressed in OSCC compared to the adjacent non-malignant tissue. They also found that the activation of TLR-2 could induce miR-146a-5p expression, causing suppression of the downstream molecule CARD10, which is recognized as a molecule regulating apoptosis and may function as a pro-apoptotic molecule by mediating the assembly of larger protein complexes in OSCC [82]. Consequently, CARD10 suppression resulted in the resistance to cisplatin-induced cell death and apoptosis in OSCC cells. Cisplatin, a commonly used chemotherapy drug, interferes with DNA replication, which kills the fastest proliferating cells [83]. Previously, TLR4 was reported to have a similar effect in OSCC activated by LPS, an endotoxin secreted by gram-negative bacteria in the oral cavity [84]. Moreover, the study on TLR-2 demonstrated that the resistance to cisplatin-induced cell death and apoptosis in OSCC cells were significantly higher in HSC3-M3 cells, a highly metastatic cell line, compared to HSC3 cells [83]. This finding indicated that OSCC cells had an increased sensitivity to TLR2 as a result of becoming malignant. Thus, TLR-2 may play an important role in OSCC progression and probably participates in the limited curative effect of chemotherapy. However, the study lacked experiments showing that oral pathogens triggered TLR-2 activation. Therefore, the exact relationship has not been established and more studies should be conducted on TLR-2.

In contrast, another oral common bacterium, *Lactobacillus plantarum*, was found to induce apoptosis in oral cancer KB cells via the upregulation of PTEN and the downregulation of MAPK signaling pathways [85].

### 3.3. Invasion and Metastasis

**Figure 3:**
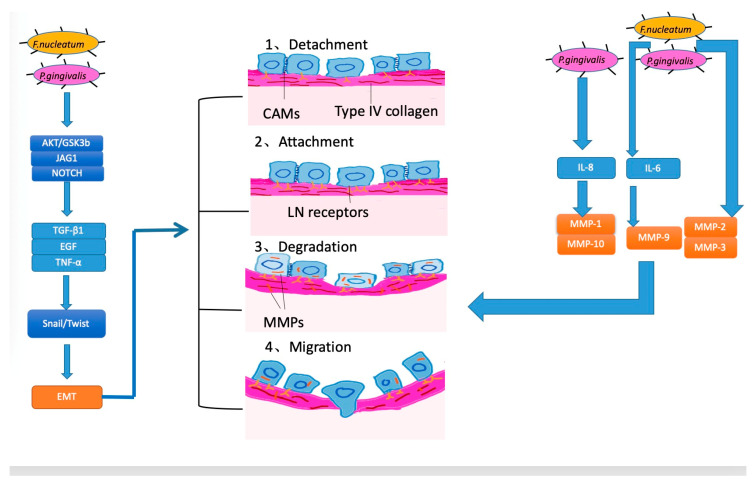
Mechanisms for how oral bacteria promote invasion and metastasis.

The mechanisms of invasion and metastasis are related to cell adhesion molecules (CAM), the extracellular matrix (ECM), and epithelial-mesenchymal transition (EMT). EMT is the process whereby epithelial cells acquire mesenchymal features and has been most studied in the processes of inflammation, invasion, and metastasis in OSCC [86]. For tumors, invasiveness and metastasis can be divided into four steps: separation from each other, increasing attachment with the basement membrane, the degradation of the extracellular matrix, and migration. According to several studies, EMT is linked to all four of these steps [87,88] ( Figure 3).

Currently, periodontal bacteria were mostly studied (Figure 3). In vitro, heat-killed *P. gingivalis* or *F. nucleatum* triggered EMT-signaling pathways in OSCC cell line cultures (H400) [46], which induced proinflammatory factors TGF-β1, EGF, and TNF-α. In this process, proinflammation cytokines TGF-β1, EGF, and TNF-α participated in a common EMT-signaling pathway, inducing the stabilization and activation of Snail. Snail and Twist are transcription factors that regulate the expression of tumor suppressors and are well-characterized regulators of E-cadherin expression [89,90]. In the former study, with the activation of Snail, E-cadherin expression decreased, which helped the cancer cells separate and attain the ability to invade and metastasize. Apart from EMT, matrix metalloproteinase (MMP), involved in the breakdown of the basement membrane and facilitation of tumor metastasis, also plays an important role in invasiveness and metastasis in OSCC, and the production of MMP along with the activation of EMT was observed in certain studies [46]. When MMPs emerge, they can dissolve the extracellular matrix and damage the basement membrane so that cancer cells can easily invade and transfer to distal locations [46,91]. Research on *P. gingivalis and F. nucleatum* reported the upregulation of MMP-2, MMP-3, and MMP-9 [46]. Furthermore, other researchers demonstrated that oral pathogens triggered TLR signaling, causing IL-6 production that activated STAT3, which induced important effectors such as cyclinD1, MMP-9, and heparinase, driving OSCC growth and invasiveness [47]. Moreover, Ha et al. infected OSCC cells with *P. gingivalis* twice a week for five weeks and found that *P. gingivalis* could stimulate MMP-1 and MMP-10 by releasing IL-8 and gingipain and increased the invasiveness of cancer cells [92].

### 3.4. Promoting Angiogenesis

**Figure 4:**
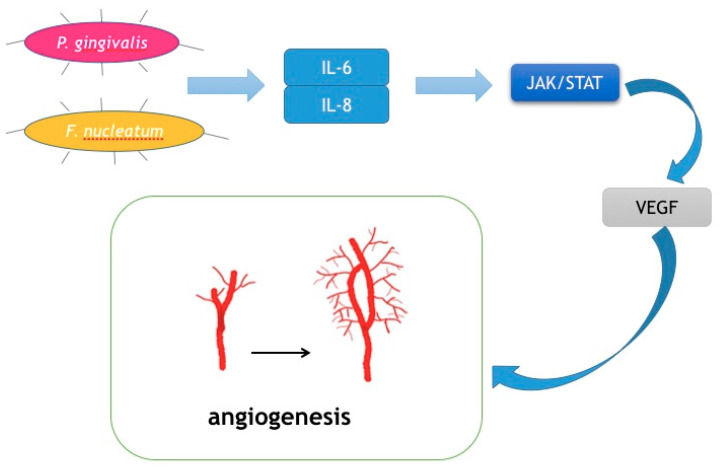
Mechanism for how oral bacteria stimulate angiogenesis.

Cancer cells are capable of fast proliferation and invasion. In a word, cancer cells are always in a hyper-metabolic state. Therefore, angiogenesis is of vital importance in cancer development. Tongue cancer is of the most common oral cancer worldwide due to the rich blood supply in tongues [93]. Vascular endothelial growth factor (VEGF) is the key mediator of angiogenesis. It stimulates irregular blood veins around cancer cells to provide nutrition and oxygen [94]. Moreover, its involvement in the differentiation and prognosis of oral cancer has been reported [95]. Recently, IL-6 was reported to induce VEGF production in OSCC [96]. Mirkeshavarz et al. designed an experiment aimed at identifying the relationship between IL-6, cancer-associated fibroblasts (CAFs), and VEGF production [96]. They cultured CAFs and oral cancer cells (OCCs) isolated from a 60-year-old male patient diagnosed with oral carcinoma separately and collectively and detected the production of IL-6 and VEGF. Their results showed that IL-6 was a factor that caused VEGF secretion in the CAF cell line and induced VEGF production in both the CAFs and OCCs. Combined with other studies, oral bacteria such as *P. gingivalis and F. nucleatum* could induce the production of IL-6. Huang et al. also found that the JAK/STAT signaling pathway participated in angiogenesis and could be activated by IL-6 [97]. IL-8 has also been reported to activate the JAK/STAT signaling pathway, which may indicate another role in OSCC progression [45]. Thus, it may be a feasible way for *P. gingivalis and F. nucleatum* to facilitate the development of OSCC, and by decreasing IL-6 and IL-8, the growth and invasion of cancer cells could be inhibited (Figure 4).

### 3.5. Assisting Cancer Stem Cells

It is well-known that OSCC patients have a low survival rate. This has been attributed to the enhanced tumorigenesis, increased invasiveness, and resistance to radiation and chemotherapy of cancer stem cells (CSC) [98,99,100,101]. CSCs are characterized by CD44, a type of membrane integrin [102]. Research conducted on oral bacteria in CSC is still lacking. However, several proinflammatory cytokines have been linked to CSCs, and oral bacteria might have an impact on CSCs as well. Patel et al. reported that cytokines in the tumor microenvironment had the ability to modulate CSC signaling pathways [103]. They reported that the increased levels of IL-6 and IL-8 in CSC samples were strongly associated with CD44, which could imply that the increased production of IL-6 and IL-8 by oral bacteria may present a favorable proinflammatory path for CSCs. A study conducted on CSCs derived from OSCC cell lines SAS and OECM-1 as well as a normal human gingival epithelioid cell line aimed to explore the cytotoxic effect of immune-modulatory proteins on OCSCs reported a tumor-suppressive effect via inhibition of the IL-6/STAT3 signaling pathway [102]. Coincidentally, the IL-6/STAT3 pathway was also involved in the process by which *P. gingivalis* and *F. nucleatum* promoted the proliferation of cancer cells [47]. Thus, *P. gingivalis* and *F. nucleatum* may stimulate the IL-6/STAT3 pathway and assist CSCs.

### 3.6. Evading Immune Attack

The immune system is an essential regulator of tumor biology with the capacity to inhibit tumor development, growth, invasion, and metastasis [104]. However, various mechanisms of tumor escape from immune attack that make it hard to eliminate tumors have been discovered by scientists [105].

In the past few years, the potential role of oral bacteria in this process has been elucidated. Our team evaluated the effect of *P. gingivalis* on the phagocytosis of Cal-27 cells (an OSCC cell line) by bone marrow-derived macrophages I and studied the effect of *P. gingivalis* on the growth of OSCC in vivo [106]. *P. gingivalis* was able to inhibit the phagocytosis of oral cancer cells by macrophages, and membrane-component molecules of *P. gingivalis* such as proteins were speculated to be the effector components. Meanwhile, sustained infection with antibiotic-inactivated *P. gingivalis* promoted OSCC growth in mice and induced the polarization of macrophages into M2 tumor-associated macrophages, which mainly displayed pro-tumor properties. These results all indicated that *P. gingivalis* could promote the immuno-evasion of oral cancer by protecting cancer cells from macrophage attack. A previous study using the squamous cell carcinoma cell line SCC-25 also verified that *P. gingivalis* could activate (B7-H1) receptors, which led to anergy and the apoptosis of activated T cells and helped cancer cells evade immune attack [76]. These experiments provide another possible mechanism for how certain bacteria promote OSCC and suggest that reducing *P. gingivalis* might be a treatment direction.

## 4. Can Oral Bacteria Be an Independent Risk Factor for OSCC?

Whether oral bacteria can be an independent risk factor is still under investigation. Nevertheless, only a few high-throughput sequencing studies on OSCC investigated the impact of other risk factors exclusive of oral bacteria [22,29]. The other risk factors included tobacco, alcohol, betel nut consumption, and HIV infection, which made it difficult to identify an independent role of oral bacteria. These other factors could contribute to compositional alterations in oral bacteria and play an indirect role in OSCC. The use of alcohol, tobacco, and betel quids was associated with a higher percentage of periodontal pathogenic bacteria in saliva [107,108,109]. Alcohol dehydrogenase 2 (ALDH2) is the most effective enzyme in the detoxification of the alcohol metabolite acetaldehyde [110], and *ALDH2* polymorphism may modify the association between alcohol use and periodontal pathogenic bacteria, and thus, affect the percentage of this kind of bacteria [109], while tobacco altered the composition of oral bacteria by causing shifts in functional pathways such as carbohydrate, energy and xenobiotic metabolism [111]. In HIV-positive patients, the community of oral microflora frequently showed noteworthy alterations [112]. The reason for this may lie in the virus itself, but the low immunity state caused by reductions in CD4+T cells in the oral cavity and the whole body accounted for most explanations [113]. Meanwhile, other factors have been confirmed as independent factors for OSCC by directly causing or promoting the cancer process. Nicotinic tobacco and betel quids could initiate the formation of reactive oxygen species, carcinogens, and genotoxicity, leading to fibroblast, DNA, and RNA damage, and finally causing oral cancer [114]. Alcohol could produce more acetaldehyde, a mutagenic and carcinogenic substance that could interfere with the synthesis and repair of DNA, produce genetic mutations, cause genotoxicity, and bind cellular proteins and DNA, finally resulting in morphological and cellular injury [115,116]. Moreover, alcohol could also reduce salivary flow, increasing the risk of cancer development [115].

Since oral cancer is associated with the use of tobacco, alcohol, and betel nuts, refining the independent role of oral bacteria seems difficult. However, epidemiological studies have reported that OSCC in a small proportion of patients who did not use tobacco, alcohol, or betel nut was closely associated with oral bacteria [117,118]. Changes in oral bacteria, such as the higher abundance of periodonto-pathogenic bacteria, could possibly result from diet and poor oral hygiene. Long-term dietary patterns and specific nutrients contributed to shaping the salivary microbiota [31]. Total dietary fiber intake was associated with an increased abundance of *Capnocytophaga* [31], which was strongly associated with OSCC [27]. The consumption of green tea or purified catechins altered the oral mucosal health and decreased the *Streptococcus* and *Staphylococcus* populations, which have been associated with tobacco users and oral squamous cell carcinoma patients [119]. Poor oral hygiene includes infrequent tooth brushing and irregular dental visits [120,121]. These bad habits could cause the overgrowth of periodonto-pathogenic bacteria and induce inflammation involved in the oncogenesis of oral cancer [111]. These findings indicate that oral bacteria, especially periodonto-pathogenic bacteria could be called “OSCC-related bacteria” and may be an independent risk factor for OSCC. However, in our opinion, defining oral bacteria as an independent risk factor is not accurate. They are distinct from drinking habits or smoking or betel nut chewing, since they represent other risk factors, which can promote cancer as well. Furthermore, inspiration can be derived from these studies in that the cancer risk could be reduced by maintaining good oral hygiene and improving the diet by consuming green tea.

In the future, more studies on the mechanisms that differentiate the role of oral bacteria from other risk factors are required. Moreover, further research investigating the involvement of LPS or proteins, symbolic substances of OSCC-related bacteria, may help clarify the role of oral bacteria. Nonetheless, certain oral bacteria either induced by other risk factors or by diet and poor oral hygiene have the ability to affect OSCC progression. Additionally, inspired by gut research, it is possible that some oral bacteria exert effects on chemotherapeutic drugs and influence the eventual curative effect [122,123]. Therefore, therapies aimed at OSCC-related bacteria can be a potential supplement to OSCC treatment.

## 5. Conclusions

During the development of OSCC, the abundance of oral bacteria changes and different bacteria show distinct alterations. Some bacteria show significantly high abundance in OSCC patients, while some bacteria make up a smaller proportion of the bacteria in OSCC tissues and present a higher abundance in healthy samples. Based on the compositional changes of oral bacteria in OSCC, a few combinations of different bacteria have been considered to be markers for oral cancer diagnosis. In OSCC, tumor development includes many mechanisms such as faster cell proliferation, escaping immune surveillance, acquiring the ability to invade and metastasize, inhibiting intrinsic cell apoptosis, promoting angiogenesis, and providing assistance to cancer stem cells. Some oral bacteria, such as *P. gingivalis* and *F. nucleatum*, participate in most pathways to assist OSCC development. Moreover, inflammation may be an agent in the process, and various inflammatory cytokines such as IL-6, IL-8, TNF-α, and PGE have impacts on the tumor microenvironment, which could aid tumor progression.

In general, this review summarizes the compositional changes in oral bacteria in OSCC reported over the past few years and the various mechanisms of how oral bacteria influence the progression of oral cancer, aimed at identifying potential methods for early diagnosis and more effective treatments.

## Appendix A

**Table A1:** Sum-up of recent 10-year research findings of noticed changing oral bacteria in oral squamous cell carcinoma (OSCC).

Year	Authors	Number of Samples	Sites of Samples	Main Findings
2012 [124]	Smruti Pushalkar et al.	10 patients	OSCC tissues and adjacent non-tumor mucosa in same patients	Species including *Streptococcus sp. oral taxon 058, P. stomatis, S. salivarius, S. gordonii, G. haemolysans, G. morbillorum, J. ignava*, and *S. parasanguinis I* were highly associated with tumor site; *G. adiacens* was prevalent at non-tumor site.
2014 [29]	Brian L. Schmidt et al.	11 patients	Cancer tissue and anatomically matched contralateral normal tissue from the same patient	Abundance of *Firmicutes* (especially *Streptococcus*) and *Actinobacteria* (especially *Rothia*) was significantly decreased relative to contralateral normal samples from the same patient.
2017 [27]	Al-Hebshi NN et al.	20 OSCC patients; 20 healthy individuals	OSCC lesions in experimental group; anatomical sites matching those affected by the OSCC lesions in control	*F. nucleatum* was the most significantly overrepresented species in the tumors followed by *P. aeruginosa* and *Campylobacter sp. Oral taxon 44*; *S. mitis, R. mucilaginosa*, and *H. parainfluenzae* were the most significantly abundant in the controls.
2017 [38]	MOK Shao Feng et al.	27 patients three groups: normal, oral potentially malignant disorders (OPMD) and cancer	swabs from the entire oral cavity in each group	Bacteria of OPMD overlap between the normal and cancer oral microbiota. *M. micronuciformis* could be an important biomarker for early oral cancer detection
2017 [22]	Wei-Hsiang Lee et al.	376 patients	saliva	Five genera, *Bacillus, Enterococcus, Parvimonas, Peptostreptococcus*, and *Slackia*, revealed significant differences between epithelial precursor lesion and cancer patients
2017 [23]	Hongsen Zhao et al.	40 patients	cancer lesion samples and anatomically matched normal samples from different patients	A group of periodontitis-correlated taxa, including *Fusobacterium, Dialister, Peptostreptococcus, Filifactor, Peptococcus, Catonella*, and *Parvimonas*, were significantly enriched in OSCC samples; *Fusobacterium* was highly involved in OSCC and demonstrated good diagnostic power
2018 [28]	Chia-Yu Yang et al.	51 healthy individuals and 197 OSCC patients	saliva	The abundance of *Fusobacterium* increased; the number of *Streptococcus, Haemophilus, Porphyromonas*, and *Actinomyces* decreased with cancer progression. *F. periodonticum, P. micra, S. constellatus, H. influenza,* and *F. alocis* were progressively increased in abundance from stage 1 to stage 4
2018 [26]	M, Perera et al.	25 OSCC cases and 27 fibroepithelial polyp (FEP) controls	25 OSCC involving the buccal mucosa or tongue (cases) and 27 Sinhala males with FEP from the same anatomic sites (controls)	Genera *Capnocytophaga, Pseudomonas*, and *Atopobium* were overrepresented in OSCC; at the species level, *C. concisus, P. salivae, P. loeschii*, and *Fusobacterium oral taxon 204* were enriched in OSCC
2018 [18]	Shun-Fa Yang et al.	103 patients	saliva	Abundance of *Firmicutes* and *Bacteroidetes* decreased
2019 [48]	Ling Zhang et al.	50 patients	tumor sites and contralateral normal tissues in buccal mucosal of same patient	*Fusobacterium, Alloprevotella* and *Porphyromonas* were enriched in cancer tissues. At the species level, the abundances of *F. nucleatum, P. intermedia* *A. segnis, C. leadbetteri,* and *P. stomatis* was significantly increased in cancer lesions
2019 [38]	Takahashi Y et al.	60 oral cancer patients and 80 non-cancer individuals as controls	saliva	*Peptostreptococcus, Fusobacterium, Alloprevotella*, and *Capnocytophaga* were more abundant in the cancer group compared to the control

Remarks: Full name of abbreviations in the table in sequence: *Peptostreptococcus stomatis* (*P. stomatis*), *Streptococcus salivarius* (*S. salivarius*), *Streptococcus gordonii* (*S. gordonii*), *Gemella haemolysans* (*G. haemolysans*), *Gemella morbillorum* (*G. morbillorum*), *Johnsonella ignava* (*J. ignava*), *Streptococcus parasanguinis* I (*S. parasanguinis* I), *Granulicatella adiacens* (*G. adiacens*), *Fusobacterium nucleatum* (*F. nucleatum*), *Pseudomonas aeruginosa* (*P. aeruginosa*), *Streptococcus mitis* (*S. mitis*), *Rothia mucilaginosa* (*R. mucilaginosa*), *Haemophilus parainfluenzae* (*H. parainfluenzae*), *Megasphaera micronuciformis* (*M. micronuciformis*), *Fusobacterium periodonticum* (*F. periodonticum*), *Parvimonas micra* (*P. micra*), *Streptococcus constellatus* (*S. constellatus*), *Haemophilus influenza* (*H. influenza*), *Filifactor alocis* (*F. alocis*), *Campylobacter concisus* (*C. concisus*), *Prevotella salivae* (*P. salivae*), *Prevotella loeschii* (*P. loeschii*), *Prevotella intermedia* (*P. intermedia*), *Aggregatibacter segnis* (*A. segins*), *Capnocytophaga leadbetteri* (*C. leadbetteri*), *Peptostreptococcus stomatis* (*P. stomatis*).
